# Upfront Xpert MTB/RIF for diagnosis of pediatric TB—Does it work? Experience from India

**DOI:** 10.1371/journal.pone.0236057

**Published:** 2020-08-05

**Authors:** Aakshi Kalra, Debadutta Parija, Neeraj Raizada, K. S. Sachdeva, Raghuram Rao, Soumya Swaminathan, Ashwani Khanna, Kamal Kishore Chopra, M. Hanif, Varinder Singh, K. R. Umadevi, K. N. Sheladia, Rama Rao, N. Vasundhara, Anil S., Nirmala A. R., Abdul Azeem, Vijay Chhajlani, Jyoti Khurana, N. J. Das, Bandana Choudhury, Sreenivas Achuthan Nair, Shalini Mall, Rajashree Sen, Sarabjit Singh Chadha, Claudia M. Denkinger, Catharina Boehme, Sanjay Sarin

**Affiliations:** 1 Foundation for Innovative New Diagnostics, New Delhi, India; 2 Central TB Division, Government of India, New Delhi, India; 3 World Health Organization, Geneva, Switzerland; 4 State TB office, Govt of National Capital Territory, Delhi, India; 5 New Delhi TB Centre, New Delhi, India; 6 Lady Hardinge Medical College and Assoc Kalawati Saran Children's Hospital, New Delhi, India; 7 National Institute of research in Tuberculosis, Chennai, India; 8 District TB Centre, Surat Municipal Corporation, Gujarat, India; 9 State TB Office, Vijayawada, Andhra Pradesh, India; 10 District TB Centre, Visakhapatnam, Andhra Pradesh, India; 11 State TB Training and Demonstration Centre/Intermediate Reference Laboratory, Bangalore, Karnataka, India; 12 District TB Centre, Indore, Madhya Pradesh, India; 13 Intermediate Reference Laboratory, Indore, Madhya Pradesh, India; 14 Office of the Jt. Director of Health Services (TB), Directorate of Health Services, Assam, India; 15 Intermediate Reference Laboratory, Guwahati, Assam, India; 16 Stop TB Partnership, Geneva, Switzerland; 17 Foundation for Innovative New Diagnostics, Geneva, Switzerland; Jamia Hamdard, INDIA

## Abstract

**Background:**

Diagnosis of TB in pediatric population poses several challenges. A novel initiative was implemented in several major cities of India aimed at providing upfront access to free-of-cost Xpert MTB/RIF to presumptive pediatric TB cases. This paper aims to describe the experience of implementing this large initiative and assess feasibility of the intervention in high TB burden settings.

**Methods:**

Data were drawn from the pediatric TB project implemented in 10 major cities of India between April 2014 and March 2018. In each city, providers, both public and private, were engaged and linked with a high throughput Xpert MTB/RIF lab (established in that city) through rapid specimen transportation and electronic reporting system. Rates and proportions were estimated to describe the characteristics of this cohort.

**Results:**

Of the total 94,415 presumptive pediatric TB cases tested in the project, 6,270 were diagnosed positive for MTB (6.6%) on Xpert MTB/RIF (vs 2% on smear microscopy). Among MTB positives, 545 cases were rifampicin resistant (8.7%). The median duration between collection of specimens and reporting of results was 0 days (same day) and >89% cases were initiated on treatment. Approximately 50% of the specimens tested were non-sputum. The number of providers/facilities engaged under the project increased >10-fold (from 124 in Q2’14 to 1416 in Q1’18).

**Conclusion:**

This project, which was one of the largest initiatives globally among pediatric population, demonstrated the feasibility of sustaining rapid and upfront access to free-of-cost Xpert MTB/RIF testing. The project underscores the efficiency of this rapid diagnostic assay in tackling several challenges in pediatric TB diagnosis, identifies opportunities for further interventions as well as brings to light scope for effective engagement with healthcare providers. The findings have facilitated a policy decision by National TB Programme mandating the use of Xpert MTB/RIF as a primary diagnostic tool for TB diagnosis in children, which is being scaled-up.

## Introduction

Tuberculosis (TB) continues to be a major public health concern especially in high-burden countries like India which accounts for approximately 28% of the 10 million global incident TB cases annually [1]. It is estimated that 8–10% of all incident cases (~15–20% in high burden countries) and 2,39,000 TB deaths occur among children below 15 years of age [2]. However, currently pediatric TB is 7% of the notified cases globally and only 6% in India [3, 4]. Of the incident pediatric TB cases estimated globally in 2014, approximately 3% and 1% have been identified as drug-resistant and rifampicin resistant (RR) cases, respectively [5].

The aforementioned proportions may grossly underestimate the actual burden of childhood TB as the diagnosis of pediatric TB is particularly challenging [4, 6]. The signs and symptoms of TB in children tend to be non-specific mimicking common childhood respiratory infections. Many children are unable to expectorate sputum adequately, and relative to adults, the likelihood of paucibacillary, smear-negative and extrapulmonary TB are high among this vulnerable population [6–8]. These challenges significantly decrease the feasibility of prompt detection of TB as well as drug resistance in children [9]. Further, following infection, the disease progresses rapidly in children, leading to significant morbidity and mortality. However, once initiated on treatment, children have a better prognosis relative to adults [10]. Together, these strengthen the importance of orienting and engaging health care providers (public and private) to suspect and test the symptomatic pediatric cases for TB. To address the gaps associated with diagnostics in pediatric TB care cascade, World Health Organization (WHO) recommends the use of a rapid diagnostic test, Xpert MTB/RIF (Cepheid Inc, Sunnyvale, CA, USA) as the initial diagnostic test in children suspected of having TB/ RR TB [11]. Based on this recommendation, a novel initiative was implemented in 10 cities of India between April 2014 and March 2018 by Foundation for Innovative New Diagnostics (FIND) in collaboration with the National TB Elimination Programme (NTEP) (erstwhile Revised National TB Control Programme (RNTCP)) of India [3]. The objectives were to provide access to quality diagnostic services to children with presumptive TB in public and private sector through provider engagement, upfront Xpert MTB/RIF testing and to build capacity of the health systems for the collection and processing of non-sputum specimens. Through this paper we aim to, i) describe the characteristics of presumptive pediatric TB cases enrolled in the initiative through the lens of upfront Xpert MTB/RIF diagnostic testing, ii) assess the operational feasibility of Xpert MTB/RIF testing, iii) assess the proportion of TB and RR (Rifampicin Resistant) TB cases diagnosed and their treatment initiation status and iv) analyse the trends in provider engagement over the total project duration.

### Initial experience from four major cities of project implementation

The pediatric TB project was initially rolled out in four major cities of India- Delhi, Chennai, Kolkata and Hyderabad. Between April 2014 to June 2016, 42,238 presumptive pediatric TB cases were enrolled through engagement of 558 public and private providers. Evidence based on data collected during this initial phase demonstrated a detection rate of 7.9% for MTB (N = 3340) and 8.8% (N = 295) for RR TB using Xpert MTB/RIF on both sputum and non-sputum specimens, a 24-hour turnaround time (between collection of specimens and reporting of results) for 95.4% of referrals, valid results for 99.7% of the cases, and operational feasibility in performing upfront Xpert MTB/RIF [12].

Although the results from the four cities were encouraging, there was a need to assess the results from the full project data across ten cities between April 2014 and March 2018. This would further aid in establishing whether the initial findings were sustained in the larger cohort in different geographies and contributes to strengthen the evidence base on Xpert MTB/RIF testing in pediatric TB population.

## Methods

Following the significant impact in the four initial intervention cities, this initiative was scaled up to six additional cities i.e. Bangalore, Guwahati, Surat, Nagpur and Visakhapatnam in 2016, and Indore in 2017. All sites were transitioned to the NTEP by the end of the project.

### Site selection and project implementation

The intervention sites were selected based on the population and estimated TB burden, as well as assessment of several factors such as lack of similar initiatives, concentration of public and private sector providers and available infrastructure and TB diagnostic services among others. These assessments were carried out by the project team, in consultation with NTEP. Subsequently, a high throughput Xpert MTB/RIF machine, exclusively dedicated for pediatric population, was installed in an existing laboratory in each city as per internationally accepted standards and WHO recommendations. The staff hired under the project at city level were given adequate training on specimen handling and Xpert MTB/RIF testing procedures. The providers from public and private sector, catering to the healthcare needs of the pediatric population, were systematically mapped at all sites. One-on-one meetings, trainings, continued medical education (CME) sessions and regular follow-ups were conducted to approach and engage providers. All the presumptive pediatric TB and DR-TB cases (0–14 years) referred from the collaborating clinics and hospitals (both public and private) were offered free of cost Xpert MTB/RIF testing. Broader coverage of the initiative was ensured through several activities including outreach programs at institutions and settings with high TB load {e.g. slums, orphanages, congregate settings, parallel government health-related programme and non-Governmental organizations (NGO’s) working in the same geographic region} and sensitization of relevant individuals in these facilities.

A presumptive *pediatric TB case* was operationally defined (as per NTEP guidelines) as a child (0–14 years) presenting with fever and/or cough for ≥2 weeks, with or without weight loss or no weight gain in the past three months, with or without history of contact with a TB case and other symptoms suggestive of pulmonary and/or extra-pulmonary TB [13, 14]. Cases who were on treatment for TB for more than one month at the time of the intervention were not tested by Xpert MTB/RIF. Providers in different regions of each city were linked to the associated Xpert MTB/RIF laboratory through a hub and spoke model. A rapid specimen transport system was established between the lab and linked public and private institutions/providers in each city to optimize operational efficacy. Feasible local transportation services acceptable to providers were utilised for this purpose including commercial courier services, local volunteers, parents/guardians of presumptive cases among others (reimbursed at standard rate). Further details have been provided elsewhere [12, 15, 16]. Both sputum and non-sputum specimens {e.g., gastric aspirate/lavage, cerebrospinal fluid (CSF), pus, lymph node aspirates, Broncho-alveolar lavage, tissue biopsies} were collected for testing under the project. Several health facilities had limited infrastructure for non-sputum specimen collection. These facilities were linked with other nearby facilities under the project where the presumptive case could be referred for specimen collection. This ensured testing of all presumptive pediatric TB cases, irrespective of the type of specimen. A rapid reporting mechanism was established to ensure that all test results were promptly communicated back to providers utilizing e-mail and short messaging service.

### Testing of specimens

The first available specimen tested on Xpert MTB/RIF was also subjected to smear microscopy using Ziehl-Neelsen (ZN) staining as per NTEP guidelines. Only Xpert MTB/RIF was performed if the volume of specimen collected was less than 1 ml in line with WHO recommendations [17–18]. Xpert MTB/RIF testing was performed as per the project diagnostic algorithm presented elsewhere [12]. Sputum specimens were tested by adding buffer in 1:2 proportion as recommended by the manufacturer (Cepheid manufacturer instructions) [19]. For non-sputum specimens, standard operating procedures (SOPs) developed by NTEP and WHO were adopted [20]. A *bacteriologically confirmed TB case* was operationally defined as having at least one pulmonary and/or extra-pulmonary specimen positive for TB by smear microscopy and/or culture and/or Xpert MTB/ RIF.

A *RR TB case* was operationally defined as bacteriologically confirmed TB case with rifampicin resistance on one or more of the following assays: Xpert MTB/RIF, line probe assay (LPA) or phenotypic drug susceptibility testing (DST) [13].

### Transition of project

The project team worked closely with the NTEP at all levels for coordinating the transition process. The initial four sites were transitioned to NTEP by 31 March 2017 as per the project work plan. Since the activities in the remaining six cities had gained significant momentum, with an increasing number of referrals and providers being engaged in each successive quarter, it was planned to stabilise and then transition these site activities and other logistics to the NTEP by the end of March 2018.

### Feasibility of Xpert MTB/RIF Implementation

The feasibility of Xpert MTB/RIF implementation was assessed in terms of the ability of the test assay to provide a valid result. The absence of a valid test result for any given assay initiated was defined as a ‘test failure’ regardless of the underlying reason. In case of ‘error’ and ‘no result’, a repeat test was performed on the remaining specimen–buffer mix. In case of ‘invalid’ and/or ‘rifampicin resistance indeterminate’ test result, repeat testing was performed on a second specimen (if available) as per the WHO recommendation [18].

The operational feasibility of offering Xpert MTB/RIF testing to presumptive pediatric TB cases through a single lab in each of the cities was assessed by analyzing the turnaround time (TAT) for specimen transportation, diagnosis, reporting of results to the providers. Transportation turnaround time (TTAT), Diagnostic turnaround time (DTAT), and Reporting turnaround time (RTAT) were defined as the duration (in days) between specimen collection and specimen receipt at the laboratory, duration between the specimen receipt and Xpert MTB/RIF testing, and duration between testing and reporting the results back to the provider, respectively. Additionally, the duration between specimen receipt at the laboratory and reporting back of results (DTAT+RTAT), and an overall turnaround time (OTAT = TTAT+DTAT+ RTAT) were also estimated.

### Data management, study variables and analysis

Data was collected for all presumptive paediatric TB and DR-TB cases by the project staff in standardized formats -through NTEP prescribed format (Annexure 15A-https://tbcindia.gov.in/showfile.php?lid=3215) in case of public sector cases and a customised referral form for private sector cases. This was entered in excel sheets specifically designed under the project and uploaded onto a web-based portal (i.e. Dropbox)[21]. Quality of data was ensured by regular scrutiny of annexes/forms with cross-validation against NTEP records by the national level project team, including verification of completeness and consistency.

For data analysis, the main variables of interest included proportion of MTB and RR cases diagnosed, positivity rates and treatment initiation status. Operational feasibility of managing specimen load was assessed by estimating turnaround time. In addition, trends in providers engagement and referrals from public and private sector were plotted over 16 quarters of the project. We used descriptive statistics {frequencies, proportions, medians, interquartile range (IQR) and range of the distributions} to describe the cohort data. Data entry, cleaning and analysis was performed using Excel 2013 and Stata, version 13 SE (StataCorp. 2013, College Station, TX: StataCorp LP).

### Ethical issues

The project was undertaken by FIND, with approval from and in collaboration with NTEP and included implementation of approved interventions under NTEP as part of Standard of TB care in India. Therefore, a separate ethical clearance was not required. During data management, care was taken to fully anonymize the data so that details provided cannot be traced back to specific individuals, or limited group of cases.

## Results

Overall, a total of 94,415 presumptive pediatric TB cases were provided access to upfront Xpert MTB/RIF testing between April 2014 and March 2018. The presumptive cases’ median age was 8 years (IQR = 4,11). Of the total presumptive cases tested, 29% were aged between 0–4 years, 33% belonged to 5–9 years age group and 38% were aged 10–14 years (Table 1). Approximately 54% of the cases were males. Majority of the cases (86%) were referred from public sector facilities.

**Table 1:** Descriptive characteristics of presumptive paediatric TB cases enrolled under the project from ten cities in India, 2014–2018 (n = 94415).

Variables	Number of pediatric presumptive cases tested N (%)	Number of MTB cases diagnosed on Xpert MTB/RIF N (%)	Positivity* %	Number of Rif resistant cases diagnosed on Xpert MTB/RIF N (%)	Positivity among diagnosed cases** %
**Total**	94415	6270	6.6	545	8.7
**Age group**					
0–4	27700 (29.3)	1157 (18.5)	4.2	72 (13.2)	6.2
5–9	30815 (32.6)	1315 (21.0)	4.3	116 (21.3)	8.8
10–14	35900 (38.0)	3798 (60.1)	10.6	357 (65.5)	9.4
**Sex**					
Female	43406 (46.0)	3807 (60.7)	8.8	328 (60.2)	8.6
Male	51003 (54.0)	2463 (39.3)	4.8	217 (39.8)	8.8
TG	6 (~0.0)	0	0	0	NA
**Sector**					
Public	81243 (86.0)	5344 (85.2)	6.6	472 (86.6)	8.8
Private	13172 (14.0)	926 (14.8)	7.0	73 (13.4)	7.9
**Smear Status**					
Positive	1711 (1.8)	1696 (27.0)	99.1	NA	NA
Negative	86649 (91.8)	4143 (66.1)	4.7	NA	NA
Not available/done	6055 (6.4)	431 (6.9)	7.1	NA	NA
**Prior H/O TB treatment**					
Yes	1830 (1.9)	881 (14.1)	48.1	155 (28.4)	17.6
No	92438 (97.9)	5389 (85.9)	5.8	390 (71.6)	7.2
Information not available	147 (0.2)	0	0	NA	NA

Rif resistant = rifampicin resistant, TG: Third gender, NA: Not applicable.

*Positivity = Number of MTB cases diagnosed on GX / Number of cases tested.

**Positivity among diagnosed cases = Number of Rif resistant cases diagnosed on Xpert MTB/RIF/ Number of MTB cases diagnosed on Xpert MTB/RIF.

Of the total presumptive TB cases tested, 6.6% (N = 6270) were positive for MTB using Xpert MTB/RIF test compared to only 2% (n = 1711) on smear microscopy. There were 15 cases positive on smear microscopy but not on Xpert MTB/RIF (14 negative and 1 error on Xpert MTB/RIF). Of 1,711 smear positive TB cases, 1,025 and 663 were low and high grade positive smears, respectively. The Xpert MTB/RIF detected 1,012 and 662 cases among low and high grade positive smears, respectively. Among cases diagnosed on Xpert MTB/RIF, positivity rate among children in the 10–14 years age group (10.6%) was observed to be more than two-fold higher than the younger age groups (4.3% in 5–9 years and 4.2% in 0–4 years). Females had approximately two-fold higher positivity rate compared to males (8.8% vs. 4.8%). Cases referred by both public and private sector facilities had similar positivity rates despite the large proportion of presumptive cases accounted for by the public sector.

Approximately 8.7% (N = 545) of MTB positive cases were found to be resistant to rifampicin on Xpert MTB/RIF. Of these, only 28% cases had a prior history of TB treatment (information was not available for majority of the cases). It was observed that RR was higher among the older age groups. The positivity rate was similar for female (8.6%) and male (8.8%) children, and public (8.8%) and private sectors (7.9%).

### Turnaround time

The mean duration of OTAT, TTAT, DTAT and RTAT were within 24 hrs with a range of 0–64 days, 0–64 days, 0–20 days, 0–33 days, respectively. The median duration for OTAT was 0 days (IQR = 0,1) and approximately 90% of the cases had their results reported within 24 hours of specimen collection (99% within 6 days). Same-day turnaround for Xpert MTB/RIF testing including specimen transportation, testing, and reporting was the norm under the project (Table 2). Additionally, the turnaround time between receipt of specimen at the lab to reporting of results was 1 day for 94% of cases (median = 0 days). The median duration between reporting of results and treatment initiation for cases who were initiated on treatment following reporting of results was 3 days (IQR = 1,6) for MTB cases (N = 5071) and 7 days (IQR = 3,14) for RR cases (N = 425). Approximately 83% of MTB cases were initiated on treatment within a week of diagnosis and 93% within 15 days of diagnosis.

**Table 2:** Project turnaround time: Time between events in the diagnostic cascade.

Variables		Days between collection of specimen and receipt (TTAT)		Days between receipt of specimen and Xpert MTB/RIF testing (DTAT)		Days between testing on Xpert MTB/RIF and reporting of results (RTAT)		Days between collection of specimen and reporting of result (OTAT)		Days between reporting and treatment initiation for TB cases*			Days between reporting and treatment initiation for Rif Resistant TB*		
	N	Median (IQR)	Mean (range)	Median (IQR)	Mean (range)	Median (IQR)	Mean (range)	Median (IQR)	Mean (range)	N	Median (IQR)	Mean (range)	N	Median (IQR)	Mean (range)
**Total**	94415	0 (0,0)	0.2(0,64)	0 (0,0)	0.2 (0,20)	0 (0,0)	0.1 (0,33)	0 (0,1)	0.5 (0,64)	5071	3 (1,6)	6 (0,466)	425	7 (3,14)	10 (0,76)
**Age**															
0–4	27700	0 (0,0)	0.1 (0,16)	0 (0,0)	0.2 (0,19)	0 (0,0)	0.1 (0,19)	0 (0,0)	0.4 (0,19)	923	2 (1,5)	7 (0,369)	48	6(2,16)	10 (0,32)
5–9	30815	0 (0,0)	0.2 (0,64)	0 (0,0)	0.2 (0–19)	0 (0,0)	0.1 (0,33)	0 (0,1)	0.5 (0,64)	1083	3 (1,6)	7 (0,368)	90	6(3,14)	10 (0,60)
10–14	35900	0 (0,0)	0.2 (0,64)	0 (0,0)	0.3 (0–20)	0 (0,0)	0.1 (0,33)	0 (0,1)	0.6 (0,64)	3065	3 (1,6)	5 (0,466)	287	7 (3,13)	10 (0,76)
**Sex**															
Female	43406	0 (0,0)	0.2 (0,64)	0 (0,0)	0.2 (0,20)	0 (0,0)	0.1 (0,33)	0 (0,1)	0.5 (0,64)	3099	3 (1,5)	6 (0,466)	263	7(3,14)	10 (0,61)
Male	51003	0 (0,0)	0.2 (0,15)	0 (0,0)	0.2 (0–19)	0 (0,0)	0.1 (0,33)	0 (0,1)	0.5 (0,33)	1972	2 (1,6)	6 (0,369)	162	6 (3,11)	10 (0,76)
TG	6	0 (0,0)	0	0 (0,0)	0.2 (0,1)	0 (0,0)	0.7 (0,2)	0 (0,1)	0.8 (0,3)	0	NA	NA	0	NA	0
**Sector**															
Public	81243	0 (0,0)	0.2 (0,64)	0 (0,0)	0.3 (0,20)	0 (0,0)	0.1 (0,33)	0 (0,1)	0.5 (0,64)	4371	3 (1,6)	6 (0,466)	375	7(3,14)	10 (0,76)
Private	13172	0 (0,0)	0.1 (0,15)	0 (0,0)	0.1 (0,17)	0 (0,0)	0.1 (0,33)	0 (0,0)	0.4 (0,33)	700	1 (1,4)	5 (0,370)	50	3 (1,8)	6 (0,44)

*for those who initiated treatment after reporting.

Rif resistant = rifampicin resistant, TG: Third gender, IQR = Inter quartile range depicted as interva.

### Specimen-wise analysis

A total of 103,045 and 91,748 specimens were tested on Xpert MTB/RIF and smear microscopy, respectively. Overall, the positivity rate on Xpert MTB/RIF was more than three-fold higher (6.6%, N = 6808) compared to smear microscopy (2%, N = 1879) (Table 3).

**Table 3:** Specimen type analysis and comparison between Xpert MTB/RIF and smear microscopy tests.

Type of specimen tested	Total specimens tested on Xpert MTB/RIF N (%)	Xpert MTB positives identified from each specimen N (% positivity)	Rif resistant identified among MTB positives N (%)	Total specimens tested on smear microscopy N (%)	Smear Positives identified from each specimen N (% positivity)
**Total**	103045	6808 (6.6)	757 (11.1)	91748	1879 (2.0)
**Sputum/Induced sputum**	**51945 (50.4)**	**3174 (6.1)**	**371 (11.7)**	**49,777 (54.3)**	**1,285 (2.6)**
**Non-Sputum**	**51100 (49.6)**	**3634 (7.1)**	**386 (10.6)**	**41971 (45.7)**	**594 (1.4)**
Gastric Aspirate/ Lavage	35866 (34.8)	1767 (4.9)	171 (9.7)	31,448 (34.3)	355 (1.1)
CSF	5714 (5.5)	353 (6.2)	32 (9.1)	3,792 (4.1)	7 (0.2)
Pus/FNAC/Lymph Node/Cervical Aspirate	3256 (3.2)	1044 (32.1)	135 (12.9)	2,227 (2.4)	163 (7.3)
		
Pleural Fluid	2850 (2.8)	111 (3.9)	16 (14.4)	2,125 (2.3)	12 (0.6)
BAL	1763 (1.7)	227 (12.9)	17 (7.5)	1,281 (1.4)	36 (2.8)
Ascitic Fluid	575 (0.6)	21 (3.7)	3 (14.3)	413 (0.5)	1 (0.2)
Tissue Biopsy	348 (0.3)	42 (12.1)	3 (7.1)	198 (0.2)	4 (2.0)
Endo-tracheal secretion/ Tracheal Aspirate	230 (0.2)	19 (8.3)	3 (15.8)	135 (0.1)	5 (3.7)
		
Pericardial Fluid	140 (0.1)	10 (7.1)	0	103 (0.1)	1 (1.0)
Others*	358 (0.4)	40 (11.2)	6 (15)	249 (0.3)	10 (4.0)

CSF: cerebrospinal fluid, FNAC: Fine needle aspiration cytology, BAL: Broncho-alveolar lavage.

*Others: Abdominal fluid, Peritoneal fluid, Abscess, Bone marrow, Bone marrow, Chyle fluid, Cystic fluid, Brain aspirate, Hip joint aspirate, Knee aspirate, Vaginal discharge, Serum, Synovial fluid, Thoracentesis, Thoracic swab, swab etc.

Of the total specimens tested on Xpert MTB/RIF, approximately 50% (N = 51,100) were non-sputum. The positivity rate for MTB for sputum and non-sputum specimens on Xpert MTB/RIF was 6.1% and 7.1% respectively, compared to 2.6% and 1.4% on smear microscopy, respectively. Among the non-sputum specimens, pus/FNAC/lymph node/cervical aspirate had the highest positivity rate (32.1%), followed by BAL (12.9%) and tissue biopsy (12.1%). Positivity rate was the lowest for pleural fluid (3.9%) and ascitic fluid (3.7%).

The Xpert MTB/RIF testing detected 11.1% (N = 757) of the specimens which were positive for MTB as RR. Over 50% of the RR positives were diagnosed on non-sputum specimens. Non-sputum specimens had similar overall positivity rate (10.6%) as sputum specimens (11.7%). Positivity rate for RR was >7% in all non-sputum specimens except pericardial fluid (0%), with the highest rate detected for endo-tracheal secretion/tracheal aspirate (15.8%), followed by pleural fluid (14.4%), ascitic fluid (14.3%) and pus/FNAC/lymph node/cervical aspirate (12.9%).

Overall, approximately 78% of the cases (N = 4869) detected in this project using Xpert MTB/RIF were of pulmonary TB of which 8.1% (N = 398) were RIF-resistant. Of the extra-pulmonary cases diagnosed (N = 1401), 10.5% (N = 147) were RR TB RIF-resistant.

### Yield of valid results on Xpert MTB/RIF

**Fig 1:**
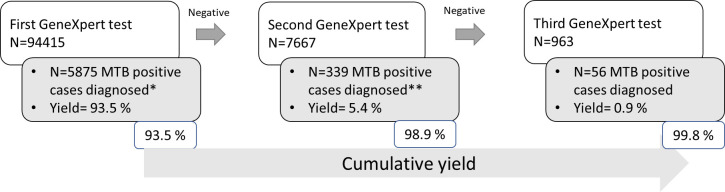
Incremental yields among first, second and third round Xpert MTB/RIF testing. * Cases diagnosed positive among those for whom the first Xpert MTB/RIF test did not show a positive MTB result but was suspected by the health care provider. **Cases diagnosed positive among those for whom the first and second Xpert MTB/RIF tests did not show a positive MTB result but was suspected by the health care provider.

Out of the 94,415 presumptive pediatric TB cases tested in the project, valid results were provided for 99.8% of cases by ensuring retesting of initial test failure (Fig 1). Of the 6270 TB cases, 93.5% were diagnosed on the first test. Repeat tests were performed for those who were negative for TB in the initial test but had persistent symptoms and were suspected to have TB by the health care provider. This yielded an additional 6.3% cases (N = 395). Of 15 smear positive but Xpert MTB/RIF negative cases, second specimen could be obtained from 6 cases all of which were negative on Xpert MTB/RIF.

### Treatment status

Of the 5,725 rifampicin sensitive cases (Table 4), 89.4% were initiated on treatment, 7.6% were lost to follow up and 1.9% died before treatment initiation and 1% were not on treatment due to other reasons.

**Table 4:** Treatment initiation status of bacteriologically confirmed rifampicin sensitive TB cases.

Variables	Total	Initiated treatment	Died	Lost to follow-up	TB case refused/not on treatment
**Overall**	5725	5120 (89.4)	113 (1.9)	435 (7.6)	57 (1.0)
**Age**					
0–4	1085 (19.0)	903 (83.2)	58 (5.3)	97 (8.9)	27 (2.5)
5–9	1199 (20.9)	1041 (86.8)	23 (1.9)	122 (10.2)	13 (1.1)
10–16	3441 (60.1)	3176 (92.3)	32 (0.9)	216 (6.3)	17 (0.5)
**Sex**					
Female	3479 (60.8)	3155 (90.7)	69 (1.9)	227 (6.5)	28 (0.8)
Male	2246 (39.2)	1965 (87.5)	44 (1.9)	208 (9.3)	29 (1.3)
**Sector**					
Public	4872 (85.1)	4414 (90.6)	110 (2.3)	301 (6.8)	47 (0.9)
Private	853 (14.9)	706 (82.8)	3 (0.4)	134 (15.7)	10 (1.2)

Among the 0–4 years age group, the proportions of cases initiated on treatment, lost to follow-up, deaths and cases who were not on treatment due to other reasons were 83.2%, 8.9%, 5.3% and 2.5%, respectively. In comparison, in the 10–14 years age group, these were 92.3%, 6.3%, 0.9% and 0.5%, respectively. There was not much difference between male and females for deaths, cases who were not on treatment, and those initiated on treatment. However, males had a higher proportion of those who were lost to follow-up compared to females (9.3% vs 6.5%). The cases from the private sector had higher proportion of lost to follow-up compared to public sector (15.7% vs 6.8%).

Among the 545 RR cases detected in this project, 468 (85.9%) were initiated on treatment, 5.1% died, 6.8% were lost to follow up before treatment initiation and 2.2% were not on any treatment due to other reasons (Table 5). More rifampicin sensitive cases could be initiated on treatment compared to those diagnosed with RR (89.4% vs 85.4%) and vice versa for number of deaths prior to treatment (1.9% vs 5.1%). There were similar proportions of lost to follow up patients among rifampicin sensitive and resistant cases with respect to different age-groups and cases coming from public vs private sectors. A higher proportion of RR males were lost to follow up compared to females (9.2% vs 5.2%). Of those cases who were lost to follow up, 83% and 78% had pulmonary TB among Rif sensitive and Rif resistant cases, respectively.

**Table 5:** Treatment initiation status of bacteriologically confirmed rifampicin resistant TB cases.

Variables	Total	Initiated treatment	Died	Lost to follow-up	Cases refused/not on treatment
**Overall**	545	468 (85.9)	28 (5.1)	37 (6.8)	12 (2.2)
**Age**					
0–4	72 (13.2)	57 (79.1)	8 (11.1)	6 (8.3)	1 (1.4)
5–9	116 (21.3)	97 (83.6)	6 (5.2)	8 (6.9)	5 (4.3)
10–16	357 (65.5)	314 (87.9)	14 (3.9)	23 (6.4)	6 (1.7)
**Sex**					
Female	328 (60.2)	287 (87.5)	18 (5.5)	17 (5.2)	6 (1.8)
Male	217 (39.8)	181 (83.4)	10 (4.6)	20 (9.2)	6 (2.8)
**Sector**					
Public	471 (86.4)	409 (86.8)	25 (5.3)	27 (5.7)	11 (2.3)
Private	73 (13.4)	59 (80.8)	3 (4.4)	10 (13.7)	1 (1.4)

### Provider engagement and referrals

**Fig 2:**
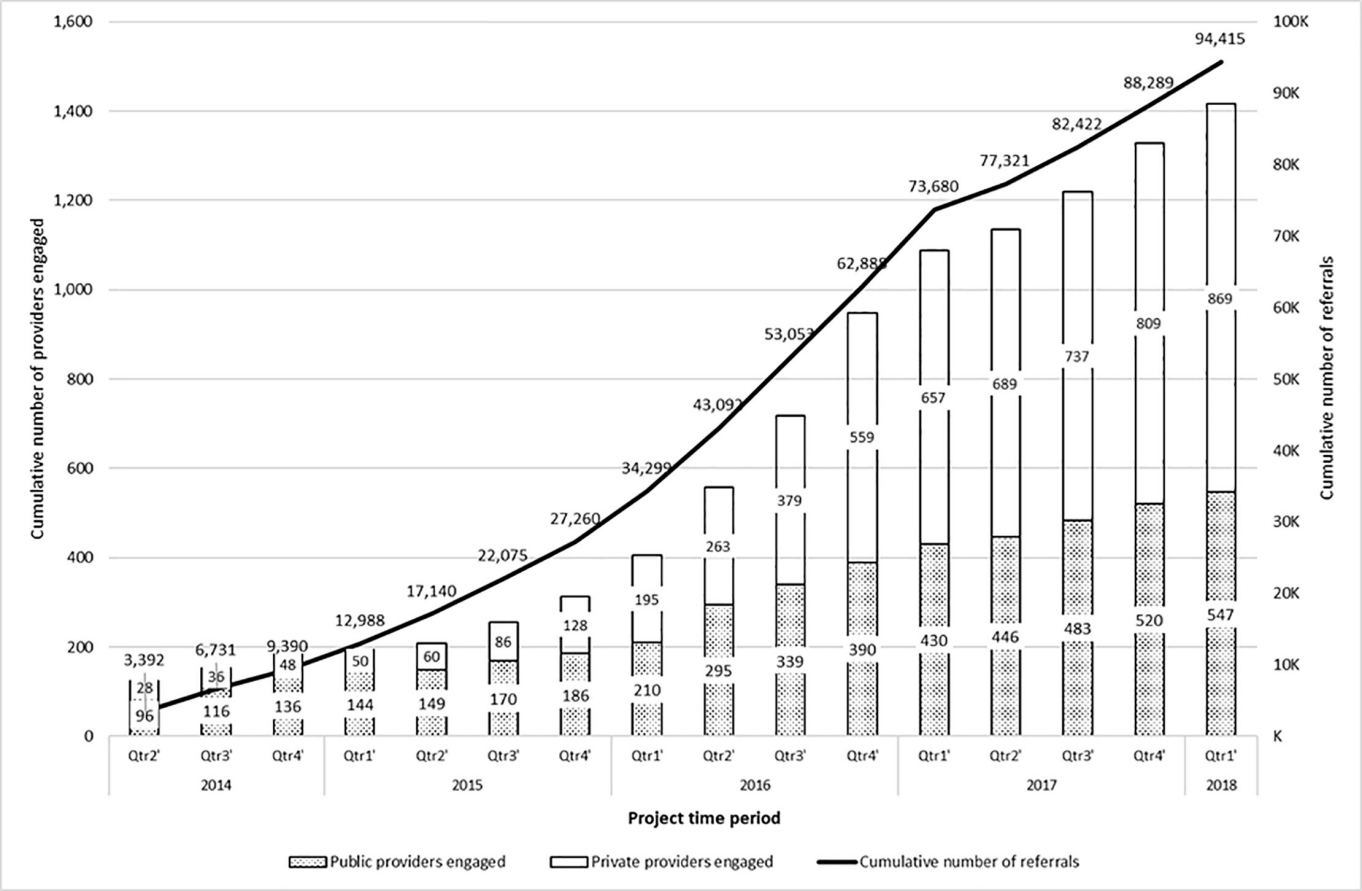
Cumulative number of public and private facilities linked and cumulative number of referrals in the project from ten cities in India, 2014–2018 (n = 94415).

The project spanned over 16 quarters between 2014 and 2018. The number of providers engaged increased from 124 (96 public sector and 28 private sector providers) in the 1st quarter of the project (i.e. 2^nd^ quarter of 2014) to 1416 (547 public sector and 869 private sector providers) in the 16^th^ quarter (i.e. 1^st^ quarter of 2018). Correspondingly, a trend towards incremental uptake of Xpert MTB/RIF testing, indicated by progressive increase in cumulative referrals, was observed during the project (Fig 2). The number of referrals increased every successive quarter from 3392 (3231 from public sector, 161 from private sector) in the 1^st^ quarter to 10,792 (8948 from public sector, 1844 from private sector) in the 12^th^ quarter of the project (until initial transition phase).

## Discussions

In this article, we have aimed to describe the experience of implementing one of the largest initiatives globally to facilitate diagnosis of TB among pediatric population, through descriptive quantitative analysis of the collected data. The central tenet of this initiative was to provide upfront access to free of cost Xpert MTB/RIF testing in programmatic settings, and the project was rolled out in ten cities of India over a span of 4 years. Our work documents several important consequences of implementing Xpert MTB/RIF testing with significant relevance to the presumptive pediatric TB cases.

First, the programmatic implementation of upfront Xpert MTB/RIF testing led to a three-fold increase in TB detection among the presumptive pediatric TB cases relative to conventional smear microscopy. This is due to the higher sensitivity of Xpert MTB/RIF for various specimens including paucibacillary ones [4, 6, 7, 9, 22]. Furthermore, Xpert MTB/RIF testing has the additional advantage of detecting RR TB cases. Our findings indicate that, through a hub-and-spoke model, the provision of upfront Xpert MTB/RIF testing can effectively address several existing diagnostic challenges in the pediatric TB population facilitating early treatment initiation.

Second, we observed differential TB detection rates among the presumptive cases based on age and sex. The number of cases enrolled as well as the proportion of TB cases diagnosed were higher in the 10–14 years age group relative to lower age groups. This may reflect increased exposure to TB cases and transmission of the disease in this school going age group. Almost similar proportion of male and female presumptive TB cases were randomly enrolled in the study. However, the positivity rate among females was almost two-fold higher than males. Secondary analysis indicated that the positivity rate among girls and boys in the 10–14 years of age-group was 14.4% and 6.8%, respectively. Interestingly, there was no difference in detection rates of RR between females and males. Evidence on existence of sex-based differences among pediatric TB cases is mixed with few previous studies from India reporting higher proportion of TB cases among female children [23–27]. Speculated reasons for this finding include neglect of the girl child in the family in terms of vaccination and nutrition resulting in poor immunity, parents lacking motivation to seek care for the girl child leading to delayed access to appropriate care, and lack of health awareness among girls due to lack of proper education [23, 25–27]. Nevertheless, it is to be noted that most studies report absolute numbers and proportions of MTB diagnosed cases received from lab reports. Furthermore, data on these cases segregated by sex of the patient are often not available. In our study, i) approximately equal proportions of female and male presumptive cases were enrolled and ii) ours is one of the first studies on pediatric population to report positivity rates based on Xpert MTB/RIF testing. Hence, albeit conducted in programmatic settings without specific sampling techniques (any engaged provider could refer a specimen), our finding that females in 10–14 years age group could indeed be a high-risk group for TB, may be valid and of policy relevance. Further studies comprehensively characterising the interrelationship between sex, age and TB diagnostics through estimation of additive and multiplicative interaction models in the pediatric population are required to confirm these findings.

Third, this project demonstrated the feasibility of maintaining same day diagnostic turn around time and high operational efficiency through implementation of Xpert MTB/RIF testing over a span of four years in the intervention cities. This was made possible with minimal human resource at the city level. One of the main reasons for increased morbidity and mortality among pediatric TB cases has been lack of or delayed access to rapid TB diagnostic tests with high sensitivity and specificity offering additional information on drug susceptibility [22, 27]. In our work, the OTAT for Xpert MTB/RIF testing including specimen transportation, testing, and reporting of results was within one day for 90% of the presumptive cases enrolled in the project. Specifically, the TAT for receipt of specimen at the lab to reporting of results (on which the project had better control, relative to transportation of specimen to the lab), was 1 day for 94% of cases. The rapid TAT was achieved by synergy of interventions such as 1) locally contextual mechanisms developed consultatively to ensure same day transportation from facilities to the city labs utilizing services of volunteers/parents/guardians/NTEP staff, 2) maintaining high throughput testing at labs to accommodate any intra-day workload surges, 3) provision of need based extended working hours, 4) 100% electronic reporting of results and 5) prompt management of technical glitches related to the lab procedures. Previous studies have reported additional gains in the diagnostic yield by testing additional specimens in cases of high clinical suspicion [28, 29]. Corroborating these findings, we documented significant gains in the diagnostic yield on testing a second specimen and a marginal gain on testing the third specimen. This indicates the incremental value of testing multiple specimens on Xpert MTB/RIF if provider has a strong suspicion.

Fourth, non-sputum specimens were quite important in diagnosis of TB in children since sputum specimens are difficult to obtain and there is significantly high prevalence of EPTB. Non-sputum specimens contributed to marginally higher TB diagnosis (7.1% vs 6.1%). For the first time under NTEP of India, both sputum and a large proportion of non-sputum specimens (about half of specimens tested), from pediatric presumptive TB cases, were tested on Xpert MTB/RIF in a routine and systematic manner. Corroborating recommendations and previous findings [28, 30, 31], Xpert MTB/RIF testing was as efficient in detecting MTB and RR TB from non-sputum specimens, as from sputum thus underlining the importance of non-sputum specimens in diagnosis of TB / RR-TB in children. The MTB positivity was >5% in majority of non-sputum specimens including precious ones such as CSF (6.2%) which is pertinent in the case of paediatric TB meningitis. We also observed that approximately 1 out of 3 specimens of pus/FNAC/lymph node/cervical aspirate was positive for MTB. This finding is of clinical relevance as swellings and lymph node enlargements coupled with positive clinical symptoms in children could be viewed as strongly suggestive of a TB case. As in previous findings, our study documented low positivity rates from ascitic and pleural fluids [32, 33]. In contrast, high RR positivity was detected in all non-sputum specimens including CSF (9.1%). Our results indicate that almost 50% of RR cases would have been missed in the absence of Xpert MTB/RIF testing of non-sputum specimens. Furthermore, since collecting sputum specimens from children is challenging in most settings, providers resort to non-sputum specimens for testing. In such cases, Xpert MTB/RIF with its high sensitivity, specificity and rapidity can provide reliable results quickly for better clinical management. This increases the significance of our project and its findings.

Fifth, our study documented high proportion of RR TB among diagnosed cases. Only 28% of these cases had a history of TB treatment with a positivity rate of 17.6%. There is indeed high likelihood of incomplete information on prior history of TB or contact with TB cases due to recall bias and stigma. Nonetheless, higher proportion of RR cases in the cohort is either suggestive of high levels of ongoing TB transmission or under diagnosis in the prevalence surveys. More studies are needed to understand the transmission dynamics and to assess whether the index case in the family was DR-TB in the treatment naïve cases.

Facilitating linkage to treatment was a mandate under this project. Around 89.4% of the Rif- sensitive and 85.9% of RR TB cases diagnosed were initiated on treatment following reporting of results. However, we also observed that 16% of MTB diagnosed cases had initiated treatment prior to reporting of results (median = 34 days, IQR = (7,89)days prior). The reason for initiation of treatment before Xpert MTB/RIF testing could not be ascertained in this project. However, this finding may be a reflection of several factors associated with care giving behaviour of health care providers such as advising Xpert MTB/RIF testing later in the diagnostic cascade for confirming their diagnosis based on other methods, or in cases who are not responding to first line treatment among others [15]. Future projects focusing on understanding care providing behaviour could provide insights into delay in utilising Xpert MTB/RIF testing.

Despite of the consistent rapid turnaround time from collection of specimens to diagnosis and reporting of results including information on Rif-susceptibility/resistance, the number of deaths (N = 141) were high. We could not confirm whether 57 of these 141 cases died prior to or after treatment initiation. Hence, these were included in the deaths prior to treatment. This way, we have underreported the number of children who were initiated on treatment and over estimated deaths in the project. However, our finding is concerning as TB is essentially a chronic disease and death results from advanced disease condition occurring in the later stages of the disease. Our results also show that most deaths occurred among children of 0–4 years age group (for both Rif sensitive and resistant TB cases) who have a higher probability of low immunity. These suggest either patient care seeking delays or provider related delays in suspecting TB and prescribing free of cost upfront Xpert MTB/RIF testing, making the need to intensify efforts at improving notification, early diagnosis and universal upfront DST imperative.

### Limitations

Our study was not a representative sample of the underlying pediatric TB population in the intervention cities. Further, many pediatric TB cases are diagnosed without microbiological confirmation (e.g., based on clinical presentation, Mantoux test) and such cases were missed by the project. Cases considered in this work were limited to those that were referred to the Xpert MTB/RIF testing lab from the sensitized providers. About 6% of the specimens could not be subjected to smear microscopy as the volume collected was less than 1 ml. However, this not likely to bias the results. Furthermore, the project could not define the target pediatric population and study accessible population in each intervention city which limits the generalizability of our findings. However, considering the limited available literature, our findings provide relevant information for policy planning. In addition, it documents the feasibility of providing upfront access to Xpert MTB/RIF testing in uncontrolled programmatic settings.

### Strengths

This intervention had several key supportive components. First, the initiative focused on targeted communication incorporating regional variation for maximizing provider and patient engagement. City specific handouts and flyers in the local language were useful in demand generation via providers and direct engagement of patients. Second, the program simplified the provider involvement process. Continued engagement of time-starved private sector providers was successful through the introduction of tactics that reduced the time needed from these providers. These included, a) an abridged referral form, b) a streamlined specimen transport, testing and reporting pathway helping in confidence-building of the providers; and c) facilitated high operational efficiency through timely reporting of results and building rapport with the providers. Third, several measures were taken to maintain trust of the engaged providers. Ensuring uninterrupted supply of cartridges and other consumables helped in ensuring rapid turnaround time and consequently gaining the provider’s trust. Preventing stock-outs required needs based estimation of cartridges needs based on usage. Based on these estimates, one-month running stock and a minimum of three months buffer stock was provided to the sites. Other laboratory consumables, as identified during the site assessment visits, were also provided and the stock was monitored in the same manner as for cartridges. Fourth, the initiative focused on creating relational capital through continuous provider engagement through regular follow-up with potential providers to seek and implement their feedback. This helped further fine tune the intervention. Fifth, timely analysis of ongoing paediatric TB practices was conducted as a basis for formulating remedial actions. For example, qualitative studies were carried out as part of the project for a) understanding how national guidelines on TB diagnosis and Xpert MTB/RIF technology have been integrated into paediatric TB care practices of different health providers, b) documenting pathways to microbiological confirmation for paediatric TB cases, and c) enabling researchers to understand current pediatric TB care practices and identify associated gaps.

The aforementioned paved the way for future course corrections incorporated into the intervention. The initial findings from the project facilitated a policy decision by India’s NTEP mandating the use of Xpert MTB/RIF as a primary diagnostic tool for TB in children. The project demonstrated the feasibility of replicating the project design in other similar settings over long periods (e.g., 4 years) of implementation time. Based on the success of the project, the intervention is now being scaled up across India by the NTEP leveraging the learnings from the project.

## Conclusions

To our knowledge, our study represented the largest cohort of pediatric cases that has been evaluated in India and one of largest global efforts which exclusively focused to implement WHO guidelines on the use of upfront Xpert MTB/RIF testing for pediatric population. Through higher MTB and RR TB detection rates, rapid turnaround times and efficiency in testing large quantities of non-sputum specimens, our findings provided evidence for the ability of a rapid diagnostic assay in effectively addressing major diagnostic challenges in presumptive pediatric TB cases. The project was able to accelerate access to improved TB diagnosis for children under 15 years of age seeking care in both the public and private sector. Additionally, the initiative was able to establish improved standards of TB care for pediatric cases by linking detected cases to a voluntary free treatment option available under NTEP, thus paving the way for public-private mix engagement.

## Supporting information

S1 Dataset (XLSB)
